# Trends in intake and outcomes for European hedgehog (*Erinaceus europaeus*) in the Czech rescue centers

**DOI:** 10.1371/journal.pone.0248422

**Published:** 2021-03-11

**Authors:** Gabriela Lukešová, Eva Voslarova, Vladimir Vecerek, Marijana Vucinic

**Affiliations:** 1 Faculty of Veterinary Hygiene and Ecology, Department of Animal Protection and Welfare and Veterinary Public Health, University of Veterinary and Pharmaceutical Sciences Brno, Brno, Czech Republic; 2 Faculty of Veterinary Medicine, Department of Animal Hygiene, University of Belgrade, Belgrade, Serbia; Sichuan University, CHINA

## Abstract

The European hedgehog (*Erinaceus europaeus*) is a species found in abundance throughout Europe. Nevertheless, it has seen a decline in some regions. This study aimed to analyze trends in intake and outcomes for hedgehogs admitted into rescue centers in the Czech Republic. In the period from 2010 to 2019, 16,967 European hedgehogs were admitted in 34 rescue centers in the Czech Republic. Most hedgehogs were admitted in September (25.30%) and October (22.14%), the fewest in March (0.96%). Most admitted hedgehogs were hoglets (59.49%). The treatment was successful in 44.39% of admitted hedgehogs; those were subsequently released into the wild. On average, they stayed in rescue centers for 48.77 days (median of 30 days). Death or euthanasia was an outcome for 25.27% and 3.15% of admitted hedgehogs, respectively. Only 0.59% of the hedgehogs remained in captivity with a permanent handicap. The highest release rate was achieved in hedgehogs admitted after falls into pits and other openings (83.19%), whereas the least success was achieved in poisoned hedgehogs (13.21%). An increasing trend (rSp = 0.9273, p < 0.01) was found in the number of hedgehogs admitted to rescue centers during the monitored period. Furthermore, not all of them required human care. Given the fact that less than a half of the admitted hedgehogs could be released, raising public awareness of this issue could help to avoid unnecessary interventions (especially in hoglets).

## Introduction

The European hedgehog (*Erinaceus europaeus*) is a terrestrial nocturnal mammal living in the area stretching from the British Isles to Western and Central Europe [1] and partly also in Northern Europe and Russia [2]. It is one of the two species of hedgehogs living in the Czech Republic and it is assumed to be more common than its relative, the Northern white-breasted hedgehog (*Erinaceus roumanicus*) [3]. The European hedgehog has been introduced to several other European islands and New Zealand [4]. Although as an invasive species it may harm local fauna [5,6], it is a natural part of the wildlands of many European countries. Hedgehogs play an important role in the regulation of the population of invertebrates, and concerning this source of nutrition, it can also be a suitable indicator of human impact on nature [7].

Although exact numbers of individuals in the population of the European hedgehog are not known, a decline in the population size has been reported in some countries [8]. The reasons for the population decline are numerous and most often directly or indirectly related to the anthropogenic activity [2]. These include a loss of natural habitats and fragmentation of the landscape [9], threats posed by domestic animals [10], road traffic [11] and others. Hedgehogs, unlike other species, seek the proximity of human dwellings due to a lower incidence of predators, namely badgers [12]. Hedgehogs also prefer overgrown lawns in gardens and orchards to forests or arable land where they cannot hide properly [7]. Thus, even though hedgehogs try to avoid direct human contact, there are benefits in staying close to humans [13]. However, along with these benefits, the risks resulting from living in an urban area also increase. Hedgehogs have to cross roads more often and consequently fall victim to road traffic [11], they may get injured by outdoor work in their nests in gardens [14] or they may fall into pits, wells and other holes dug by humans from which they cannot escape without help.

Hedgehog populations are not solely affected by anthropogenic activities. There are many non-anthropogenic factors which also affect the health of hedgehogs, such as parasites [15]. A healthy and strong hedgehog can tackle these but for individuals that are injured or stressed, for example due to captivity, it can be an aggravating factor. In summer, myiasis can occur when fly larvae infest open wounds which impairs the health of hedgehogs and their ability to heal [16]. Rearing of hoglets is also a critical period. It is not entirely clear how many litters the European hedgehog has in a year. In milder climatic conditions, they can successfully rear two litters in a year [17]. The number of litters can affect the survival prospects of both the young and the female during winter. They have to build up good fat reserves to survive the winter hibernation. The hoglets from late litters may grow faster than those born earlier to prepare for the winter season [18]. The duration of hibernation is related to climatic conditions and may be shorter in milder ones [19]. The age of hedgehogs is somewhat problematic to determine. Nevertheless, it can be estimated based on some physical parameters and weight [20].

Rescue centers are involved in the protection of European hedgehogs as they provide care for disabled individuals. The hedgehogs are admitted to the centers due to injury, disease, or because they are orphaned young that will not survive without human help. The care itself includes an evaluation of the health condition and handicap with which the hedgehog was admitted to the center, veterinary treatment, feeding and ensuring a suitable temperature of the environment [21]. The main goal of rescue centers is to return the animal to its natural habitat. Thus, it is important to correctly determine whether the animal can survive on its own. The success depends on the assessment of the animal’s health, along with an examination of dental calculus and a parasitological examination [22]. A very important aspect is the choice of the most suitable location for releases in terms of safety and food sources without endangering the existing population of animals on the location. Hedgehogs can be subjected to a variety of routine examinations as well as surgeries [23], but the benefits to the animal itself should always be weighed with particular care concerning the quality of life, the possibility of returning the animal to the wild and the onset of stress during the stay in the rescue center. The data obtained from rescue centers can contribute to the protection of wild species in a given region, to the understanding of the impact of anthropogenic activity on endangered species, and can serve as one of the tools of preservation of wild species of animals.

The aim of the study was to evaluate the number of European hedgehogs admitted to rescue centers in the Czech Republic in the period from 2010 to 2019, taking into account the reasons for their admission and outcomes. The study also aimed to analyze the length of stay of hedgehogs in the rescue centers in connection with the cause of admission and the percentage of hedgehogs that could be released back into the wild. The study intended to determine factors affecting European hedgehogs in the wild, namely factors leading to their admission to the rescue centers. Knowledge of these factors is important when proposing mitigation measures. The analysis of trends in intake and outcomes for European hedgehogs including the reason for admission and length of stay is beneficial also for rescue center operators.

## Materials and methods

The data on European hedgehogs admitted to rescue centers in the Czech Republic was obtained from the records of the Ministry of the Environment that collects data from the National Network of Rescue Centers (comprised of 34 rescue centers). Any rescue center is obligated to provide each found handicapped animal with a complex care including first aid, veterinary treatment, therapy and rehabilitation. According to Czech legislation, a handicapped animal is a wild animal that is temporarily or permanently unable to survive in the wild due to injury, illness or other factors [24]. The rescue center records include information on all animals admitted to the rescue centers in the Czech Republic including the reason and date of admission and the outcome for each individual animal.

The subjects of this retrospective study were all European hedgehogs admitted to the rescue centers in the Czech Republic from January 1, 2010, to December 31, 2019. For the purpose of statistical evaluation, the numbers of hedgehogs admitted in individual years and months (for those where the month of admission was recorded) of the monitored period were calculated. Furthermore, hedgehogs were divided into groups according to the reasons for admission to the rescue centers and the outcomes. For hedgehogs with known admission and discharge dates, the length of stay was also calculated and evaluated.

The categorization of the admission and outcome groups used in this study corresponds to the guidelines for the evidence of animals in rescue centers in the Czech Republic. The categories of reasons for admission are described in Table 1. All hoglets were recorded in the ‘Hoglet’ category; other categories included only adult hedgehogs. The categories of outcomes are described in Table 2. Each animal was recorded only in one admission and one outcome group.

**Table 1:** Reasons for admission of European hedgehogs to the rescue centers in the Czech Republic.

Reason for admission	Description	Example
**Hoglet**	All hoglets	Abandoned (genuinely or supposedly), orphaned, injured, exhausted, underweight hoglets
**Exhaustion, starvation**	Underweight and dehydration, no symptoms of infection	Hedgehogs showing signs of dehydration, apathy
**Road and rail traffic**	Hedgehogs injured by road or trail traffic	Injured hedgehogs found on the side of the road
**Awakened hibernator**	Interrupted hibernation	Hedgehogs found active during daylight in winter season, not in the nest
**Injury by another animal**	Hedgehogs suffering from a bite wound	Hedgehogs admitted after being seen to get bitten e.g. by a dog
**Infections, parasites**	Hedgehogs showing the symptoms of infection (including parasite infection)	Symptoms include sneezing, nasal discharge, diarrhea, severe flea infestation
**Falls into pits**	Hedgehogs that fell down into pits, holes and other openings, unable to escape by themselves	Hedgehogs found in pits etc.
**Injuries**	Hedgehogs suffering from an injury other than a bite wound	Fractures, burns, paralysis, gardening-related injuries
**Entanglement**	Hedgehogs trapped in some way	Hedgehogs found tangled in nettings, trapped in discarded plastic waste
**Intrusion**	Hedgehogs that invaded a building, namely when they could not get out and were in danger	Hedgehogs found in a garage
**Unnecessary capture**	Hedgehogs brought into the rescue for no apparent reason	Healthy, well fed hedgehogs showing no signs of injuries or diseases brought into the rescue center by a member of the public
**Takeover**	Hedgehogs kept or born in captivity handed in to the rescue centers	Hedgehogs kept by someone in captivity including those born in captivity handed in to the rescue center (not captured in the wild before admission)
**Rescue transfer**	Hedgehogs found in a dangerous place and kept for observation/treatment	Hedgehogs found in the nest near a road
**Weather extremes**	Hedgehogs admitted to the rescue centers due to extreme weather	Hedgehogs saved from nests destroyed by a flood
**Poisoning**	Hedgehogs showing signs of poisoning	Hedgehogs showing symptoms of different types of poisoning, no external injuries
**Other**	Reasons other than those described above	E.g. oil contamination, confiscation of illegally kept hedgehogs

**Table 2:** Outcomes for European hedgehogs in the rescue centers in the Czech Republic.

Outcome	Description
**Release**	Hedgehogs that recovered and were released back to their natural habitat
**Death**	Hedgehogs that died in the rescue center (unassisted death)
**Euthanasia**	Hedgehogs euthanized for health reasons by a veterinarian
**Permanent captivity**	Hedgehogs that could not be released back to the wild and had to remain in permanent care
**Escape**	Hedgehogs that escaped or got lost during rehabilitation
**Unknown**	Not specified

The length of stay of European hedgehogs in the rescue centers was calculated as the difference between the date of admission and the date when the stay of a hedgehog in the rescue center was terminated. Hedgehogs for which the dates were not recorded were excluded from this analysis as it was not possible to determine their length of stay. For hedgehogs that were admitted on the same day as their record was terminated, the length of stay was set at 0.5 days. It is an estimate of the time needed to examine the animal and decide upon its future (euthanasia, release after treatment, etc.). The average length of stay was calculated separately for each outcome group.

The data was evaluated using the statistical program UNISTAT 6.5 for Excel (Unistat Ltd., London, UK). To assess the development of the number of recorded European hedgehogs in rescue centers over the years, the Spearman correlation coefficient was used to determine the coefficient of order correlation. A chi-squared test with Yates’s correction for continuity within the methodology of 2x2 contingency tables was used for statistical evaluation of the frequency differences within individual groups with numbers of individuals > 5. The Kruskal-Wallis ANOVA (and subsequently multiple comparisons for t-distributions) was used for statistical comparison of the length of stay between the individual categories according to the method of termination of the stay in rescue centers. In the tests used, the value of p < 0.05 was determined to be statistically significant.

## Results

**Fig 1:**
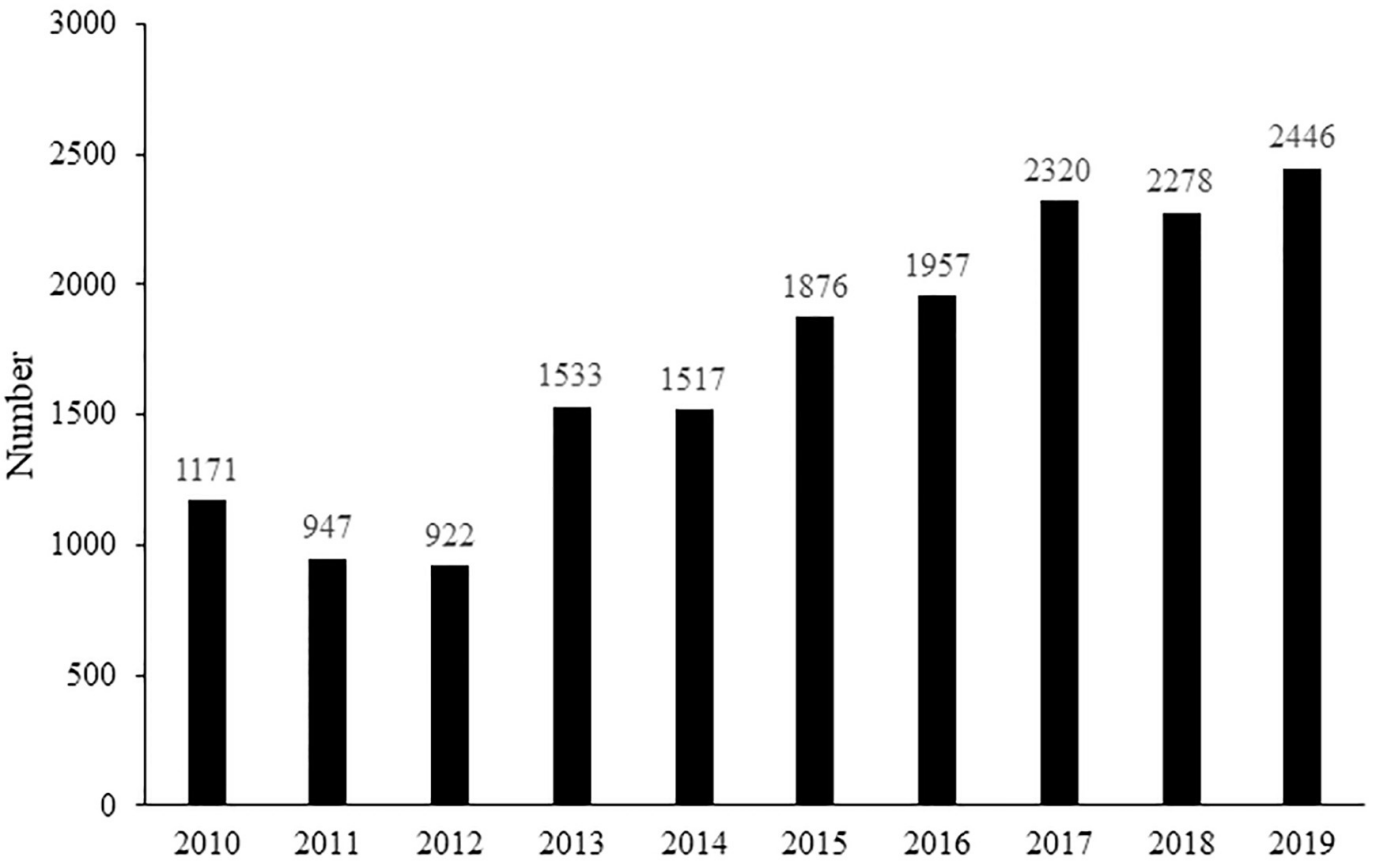
The number of European hedgehogs admitted to rescue centers in the Czech Republic in the period from 2010 to 2019.

During the period from 2010 to 2019, a total of 16,967 European hedgehogs were admitted to 34 rescue centers in the Czech Republic. Fig 1 shows the number of hedgehogs admitted in each year of the monitored period. A rising trend was found in the number of European hedgehogs admitted to the rescue centers in the Czech Republic (rSp = 0.9273, p < 0.01).

The number of European hedgehogs admitted to rescue centers in individual months in the period from 2010 to 2019 is shown in Table 3.

**Table 3:** Number and percentage of the total number of European hedgehogs admitted to rescue centers by individual months in the period from 2010 to 2019.

Month	Admitted hedgehogs (n = 16,784)	
	number	%
January	421	2.48^g^
February	227	1.34^i^
March	163	0.96^j^
April	274	1.61^h^
May	398	2.35^g^
June	610	3.60^f^
July	677	3.99^f^
August	2,248	13.25^d^
September	4,292	25.30^a^
October	3,756	22.14^b^
November	2,535	14.94^c^
December	1,183	6.97^e^

^a-j^The values with different superscript letters in a column are significantly different (p < 0.05).

Most hedgehogs were admitted in autumn months (September, October, November), fewer in August and December. The lowest numbers of hedgehogs were admitted at the end of winter and in the spring (March, February and April).

The reasons for admission of European hedgehogs to rescue centers are given in Table 4.

**Table 4:** The number and percentage of the total number of European hedgehogs admitted to rescue centers in the years 2010 to 2019 according to the reason for their admission.

Reason for admission	Admitted hedgehogs (n = 16,967)	
	number	%
Hoglet	10,170	59.49^a^
Exhaustion, starvation	1,558	9.18^b^
Road and rail traffic	614	3.62^f^
Awakened hibernator	674	3.97^d^^,^^e^^,^^f^
Injury by another animal	728	4.29^d^^,^^e^
Infections, parasites	332	1.96^g^
Falls into pits	232	1.37^h^
Injuries	742	4.37^d^
Entanglement	36	0.21^i^^,^^j^
Intrusion	51	0.30^i^
Unnecessary capture	623	3.67^f^
Takeover	21	0.12^j^^,^^k^
Rescue transfer	24	0.14^j^
Weather extremes	30	0.18^j^^,^^k^
Poisoning	53	0.30^j^
Other	1079	6.36^c^

^a-j^The values with different superscript letters in a column are significantly different (p < 0.05).

Most European hedgehogs were admitted to rescue centers as hoglets (59.49%). Other reasons for admission were recorded in less than 5% of hedgehogs, with the exception of exhausted and starving animals (9.18%) and other reasons, which were not specified (6.36%).

The outcomes for European hedgehogs admitted to rescue centers are given in Table 5.

**Table 5:** The number and percentage of the total number of European hedgehogs admitted to rescue centers in the period from 2010 to 2019 according to the outcome.

Outcome	Hedgehogs (n = 16,967)	
	number	%
Release	7,531	44.39^a^
Death	4,287	25.27^b^
Euthanasia	535	3.15^e^
Permanent captivity	99	0.59^f^
Escape	11	0.06^g^
Unknown	1,493	8.79^d^
Still treated	3,011	17.75^c^

^a-g^The values with different superscript letters in a column are significantly different (p < 0.05).

Almost half (44.39%) of the European hedgehogs could be released back into the wild. Less than a third of the admitted hedgehogs died or had to be euthanized.

A total of 3,011 (17.75%) hedgehogs were still being treated in the rescue centers by the end of the monitored period and thus are not included in this analysis.

**Fig 2:**
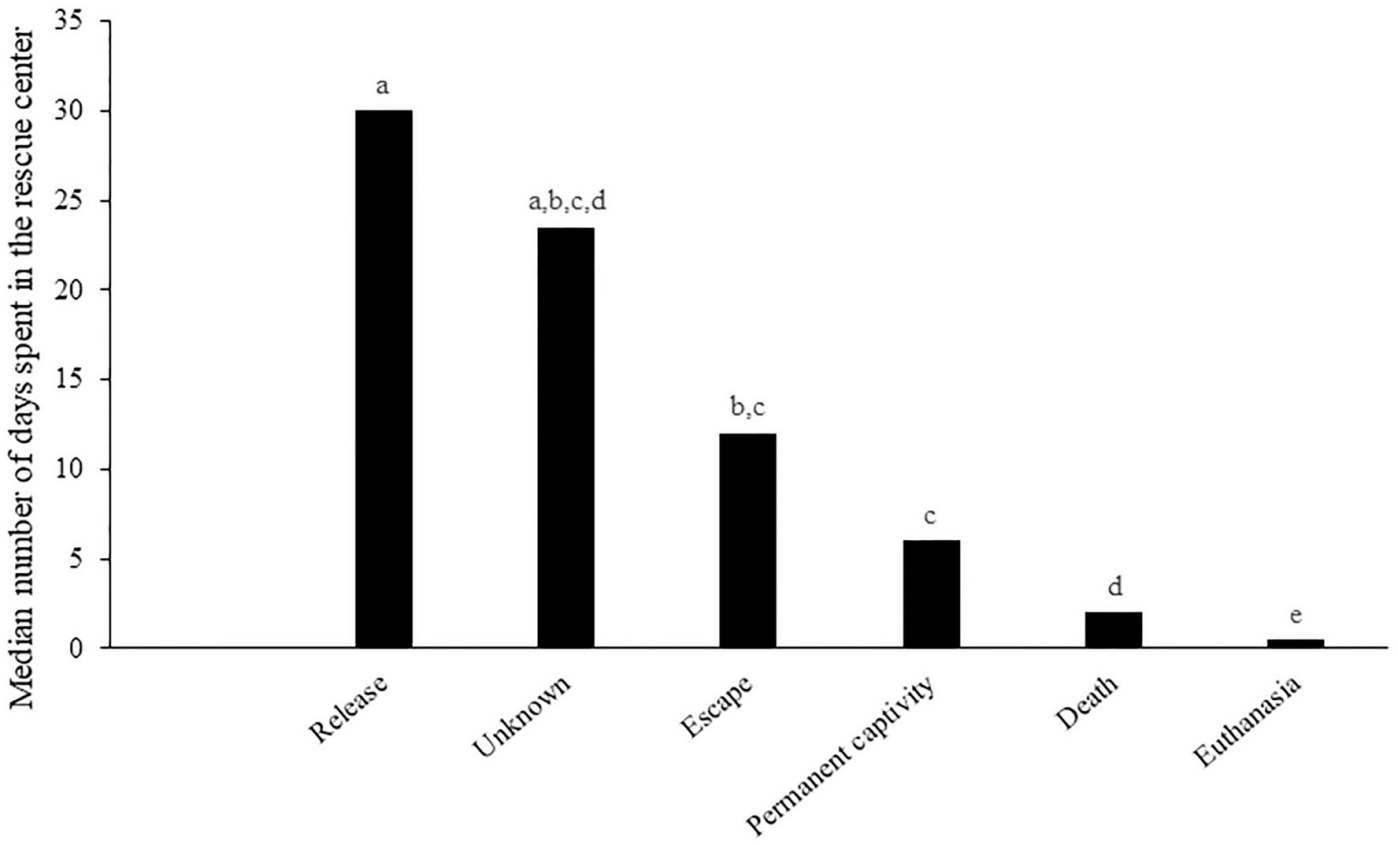
Comparison of the length of stay of European hedgehogs in rescue centers depending on the outcome. ^a-e^The values in columns with different superscript letters are significantly different (p < 0.05).

A comparison of the length of stay of European hedgehogs in rescue centers depending on the outcome is shown in Fig 2.

The hedgehogs that had to be euthanized had the shortest length of stay in the rescue centers (median of 0.5 days). Contrarily, the groups of animals subsequently released into the wild and animals with an unknown outcome spent the longest time in the rescue centers (median of 30 and 23.5 days, respectively).

**Fig 3:**
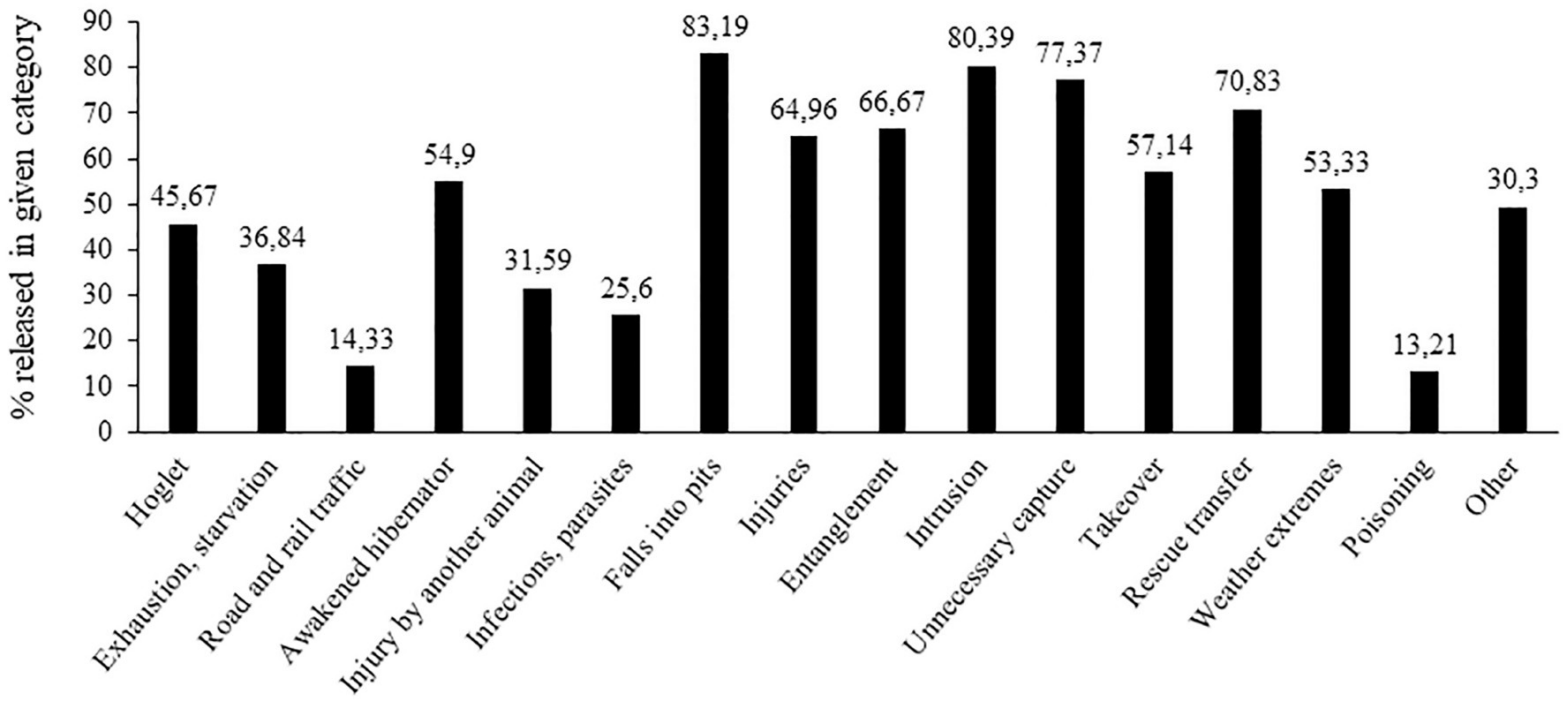
The number of European hedgehogs released into the wild depending on the reason for admission to rescue centers.

The number of released European hedgehogs in relation to the reason for their admission to rescue centers is given in Fig 3.

Fig 3 shows that hedgehogs admitted after falling into pits and other places from which they were unable to get out on their own were most often released back into the wild (83.19% of hedgehogs admitted for this reason were subsequently released). Also hedgehogs admitted due to intrusion could be subsequently released in most cases (80.39%). On the contrary, the lowest release rates were achieved in hedgehogs admitted due to poisoning (13.21%) and after a collision with road or rail traffic (14.33%).

## Discussion

The number of European hedgehogs admitted to rescue centers in the Czech Republic increased significantly during the period from 2010 to 2019. This fact may indicate that the awareness and willingness of people to deliver handicapped hedgehogs to rescue centers are gradually increasing. Alternatively, the higher numbers of admitted hedgehogs may be a result of an increasing number of injured hedgehogs due to expanding traffic, evolving anthropogenic activity and other factors. Due to the absence of data on the population of European hedgehogs and the numbers of handicapped hedgehogs (including those not reported), this cannot be clearly assessed. Both scenarios are possible. On the one hand, negative effects of global change on animal populations have been increasing in the recent decades [25]. On the other hand, the public is more concerned about the environment, protection and rescue of wild animals than ever before, and a lot of wildlife conservation organizations have been established recently.

A total of 16,967 European hedgehogs were admitted to rescue centers in the Czech Republic during the monitored period. The data in similar studies differ according to the number of rescue centers included and according to the observed period. Grogan and Kelly [26] reported 754 western hedgehogs admitted to four rescue centers in England from 2000 to 2004, and Molina—Lopéz et al. [27] reported 1,309 hedgehogs admitted to one rescue center in Catalonia. Crespo et al. [28] reported 490 hedgehogs admitted to rescue centers in Valencia, Spain, between 2009 and 2013, 84% of which were European hedgehogs and most of them were admitted during the summer and autumn with a decline in winter. This admission distribution is similar to our results, which show a sharp increase in the number of hedgehogs admitted in September, October and November with a decline in the winter months. The increase is related to the admission of hoglets and also hedgehogs found outside their nests in late autumn, when they should be hibernating [29]. In contrast to the study by Fowler and Racey [29], there was no increase in the number of admitted hedgehogs in winter in our study. The increased admission of hedgehogs in winter may be related to the issue of more than one litter of European hedgehogs in milder climatic conditions [17]. Saboreau and Dotourné [30] reported two mating seasons during one year in France according to the activity of male hormones in hedgehogs. Hoglets from the second litter may not be able to put on enough weight in time. The higher number of admissions in the winter months (November and December) is therefore associated with a critical period of hibernation. Hedgehogs that do not have enough body weight to survive hibernation are still foraging in winter [31]. Robinson and Routh [21] reported that hedgehogs weighing less than 450 g in the fall or those moving about out of their nests during the day with symptoms of weakness or a health disorder may need some help to survive the winter. However, a different optimum weight before hibernation was reported in other studies. According to Bearman-Brown et al. [32], the optimum weight of hedgehogs before hibernation is at least 600 g. The issue of underweight hedgehogs not surviving winter is well known to the public, which is why hedgehogs are often brought to rescue centers in this season. Similarly, Garcês et al. [33] reported the most common reason for admission in Portugal to be weakness (30.7%). Hedgehogs may go through periods where they are unsuccessful in finding enough food. However, feeding wild hedgehogs is a controversial issue. Soft food intended for dogs and cats is often offered, which can contribute to the formation of dental calculus in hedgehogs, or the provision of such easily accessible food may disrupt the hibernating behavior of hedgehogs [34]. On the other hand, these sources can help hedgehogs reach optimum condition before winter hibernation or be an alternative source of food at the time when their primary food is less available. Reeve and Huijser [35] also came to similar conclusions when analyzing data from British rescue centers from 1992 to 1998. They found the highest number of admissions of European hedgehogs from July to December. In Portugal, 740 western hedgehogs were admitted to two rescue centers [33] in the period from 2002 to 2019. There, besides the high admission rates in the summer, an increased intake was also found in the spring months. On the contrary, we found the number of admissions of European hedgehogs in the spring to be the lowest. This probably shows the effect of different habitats and climate on the ecology of hedgehogs, which also has an impact on the length of hibernation and reproductive cycle [36]. Particularly, a shorter hibernation period may be the reason for more hedgehogs admitted in the spring in southern countries than in the Czech Republic. Hedgehogs that do not hibernate are more likely to be harmed and admitted to rescue centers during this period than hedgehogs that do not stop hibernating until late spring in the Czech Republic. The impact of climate on hibernation and related reproduction as well as the effects of sudden changes were also studied in other animal species [37,38].

The numbers of European hedgehogs admitted to rescue centers for different reasons may vary depending on the selected criteria on which the authors based the classification of animals into individual admission groups. In the Czech Republic, the most frequently recorded reason for admission of a handicapped hedgehog was being a hoglet (59.49%). In contrast to our results, Crespo et al. [28] reported only 14% of hedgehogs admitted as hoglets to three rescue centers in Spain. Many hoglets may be brought to rescue centers unnecessarily without the need of human care. The problem of removing healthy animals (e.g. hoglets) from their natural habitat due to concerns about their health, although such concerns are out of place and are driven by public ignorance, is also documented in other animal species [39]. The disadvantage of hoglets admitted to rescue centers may be their minimal experience of living in the wild, which may have a negative effect on their survival after release into the wild the following spring [22]. However, some studies have shown that hedgehogs that have spent their first winter in a rescue center can forage and build nests after release as well as reproduce without difficulty despite the lack of previous experience in the wild [40,41]. Bunnel [18] found that hoglets from late litters did not have a reduced chance of surviving the winter than hoglets born earlier in the season. However, hoglets in rescue centers including those that were admitted in good health condition have a relatively high mortality as shown by mortality statistics. Contrary to the original intentions, the chances of survival can be reduced by delivering a hoglet that does not need human care to a rescue center [42]. Nevertheless, human help is needed in some cases. Hoglets born in the first litter of a young female are especially vulnerable. The young female hedgehogs do not have enough experience and their offspring are more likely to die [17]. In such cases as well as in orphaned hoglets, early intervention can help them to survive. Each case must be assessed properly and unsubstantiated interventions should be avoided. Unfortunately, people tend to act on the basis of their emotions especially when their knowledge of wildlife is limited. Young animals found on their own are often considered abandoned even if actually they are not. Also fawns and leverets that just wait in hiding until their mothers return [43] are often taken from the wild by well-meaning people. Many rescue centers and conservation organizations carry out projects aimed to educate the public about wildlife to address this issue.

Anthropogenic factors are a very common cause of admission of animals to rescue centers [27]. In our study, anthropogenic interferences were involved in admission of hedgehogs that were unnecessarily captured, fell victim to collisions with road or rail traffic, fell into pits, wells and other holes dug by humans, invaded a building or were trapped in netting. Collisions with road or trail traffic are frequently reported as a reason for death or injury in hedgehogs [44]. In our study, 3.62% of hedgehogs were admitted to rescue centers as a result of vehicular traffic. Collisions with road or rail traffic are often fatal for hedgehogs or serious injuries to the limbs and jaws occur [16]. The numbers of hedgehogs run over on roads varies depending on the time and the place where the road passes. Due to hedgehogs’ peak activity during summer, the most road collisions and fatal incidents occur in summer, more frequently in urban areas and on sites with dense vegetation along roads and rails [11,44]. The movement of hedgehogs in such areas is related to the availability of food and presence of predators [45]. Males are thought to travel greater distances during the night, which is probably related to their search for a suitable mate [46,47]. Hedgehogs occupying gardens can be attacked by domestic animals, namely dogs [10]. In our study, 4.29% of hedgehogs were brought to rescue centers after being injured by another animal. Knowledge of anthropogenic factors responsible for temporary or permanent incapacitation of wild animals is important for understanding the threats to wildlife [48]. Subsequently, attempts are made to reduce the risks. Wildlife crossings are built to allow animals to cross the road safely [49]. Concerning the risks related to domestic animals and pits as well as unnecessary capture, prevention and public education are essential.

Ideally, after a successful treatment provided by the rescue center, a healthy hedgehog is released back into its natural habitat. In our study, less than a half (44.39%) of hedgehogs admitted to the rescue centers in the Czech Republic was subsequently released back into the wild. The highest release rate was achieved in hedgehogs admitted to rescue centers after falling into pits and other openings and due to intrusion. Garcês et al. [33] reported a 66.6% release rate at two rescue centers in Portugal. Crespo et al. [28] reported even higher release rate (69%) for hedgehogs admitted to rescue centers in Spain. In their study, the best-rated groups in terms of release were hedgehogs randomly caught and hoglets. In our study, only 45.67% of hoglets were released back into the wild. Low release rates were found in hedgehogs admitted with infections and parasitic infestations and after a collision with a road or rail traffic. Crespo et al. [28] reported that only 35% of hedgehogs suffering from infections or parasites and 17% of hedgehogs admitted after a collision with road traffic could be subsequently released. Their results are consistent with our study. In the Czech Republic, 25.60% of hedgehogs admitted with infections and parasitic infestations and 14.33% of hedgehogs admitted after a collision with road or rail traffic were released into the wild. A higher release rate was found in hedgehogs admitted due to other injuries; 64.96% of them were returned back to the wild. The severity and extent of injuries affect the treatment and prognosis [26]. The lowest release rate was achieved in poisoned hedgehogs in our study. Only 13.21% of poisoned hedgehogs admitted to rescue centers in the Czech Republic could be subsequently released. Research has shown that poisoning by industrial chemicals, including rodenticides, may have been a contributory factor to the decline in hedgehog populations in some areas. Dowding et al. [50] found that the European hedgehog has similar rates of exposure to anticoagulant rodenticides to those of specialist predators of small mammals.

Keeping wild hedgehogs in captivity is disputable with regard to their welfare [51]. Thus, the low percentage of hedgehogs remaining in captivity due to a permanent handicap found in our study is encouraging. However, a successful treatment followed by releasing hedgehogs back to their natural habitat does not ensure their subsequent survival in the wild. According to Molony et al. [52], the ability of released individuals to adapt to novel sites is critical. Many hedgehogs cannot be returned to the area where they were found. The right release site has to be chosen. Releasing hedgehogs in urban areas is questionable. They seem to prefer such sites but there is also an increased risk of collisions with road traffic and other anthropogenic activities [53]. Prior to release, it is necessary to consider welfare of the individual as well as protection of the wild population and choose the release site accordingly [54].

The time spent in a rescue center by individual hedgehogs ranged from 0.5 to 1,261 days. The shortest length of stay in rescue centers was found in hedgehogs that died (median 2 days) or had to be euthanized (median 0.5 days). Similar results were reported by Molina et al. [27] for a rescue center in Catalonia. Molina et al. [27] found the median length of stay for all animals to be 9 days, 17 days for subsequently released animals, 3 days for animals having died, and 0 days for euthanized animals. In contrast to their study, hedgehogs spent a longer time (median 30 days) in rescue centers in the Czech Republic before release. The time the hedgehogs spent in the rescue center is related to the time needed for animals to achieve optimum condition and thus maximize their chances of survival [55]. According to Molony et al. [52], temporary captivity improves the chances of survival by allowing the build-up of fat reserves and reducing manipulation stress suffered on release.

## Conclusion

Rescue center records provide information on factors that endanger the health and life of European hedgehogs and contribute to the decline in their populations in the wild. The knowledge of the reasons leading to hedgehogs’ inability to survive in the wild must be taken into account when planning conservation measures to reduce these risks. By treating hedgehogs in rescue centers, it is possible to mitigate the impact of human activity, enable handicapped animals to return to the wild and support on-site conservation efforts. In the monitored period, about a half of the admitted hedgehogs were released back into the wild. An increasing trend was found in the number of hedgehogs admitted to the rescue centers; however, not all of these hedgehogs required human care. In this regard, educational activities are important to increase the public’s ability to distinguish between animals in need of human care and those that are healthy and independent to avoid unnecessary interventions (especially in hoglets).
